# Antitumoral Effect of Laurinterol on 3D Culture of Breast Cancer Explants

**DOI:** 10.3390/md17040201

**Published:** 2019-03-29

**Authors:** Sara García-Davis, Ezequiel Viveros-Valdez, Ana R. Díaz-Marrero, José J. Fernández, Daniel Valencia-Mercado, Olga Esquivel-Hernández, Pilar Carranza-Rosales, Irma Edith Carranza-Torres, Nancy Elena Guzmán-Delgado

**Affiliations:** 1 Facultad de Ciencias Biológicas (FCB), Universidad Autónoma de Nuevo León (UANL), Av. Pedro de Alba s/n, 66450 San Nicolás de los Garza, Nuevo León, México; sara.garciadv@uanl.edu.mx (S.G.-D.); jose.viverosvld@uanl.edu.mx (E.V.-V.); 2 Instituto Universitario de Bio-Orgánica Antonio González (IUBO AG), Centro de Investigaciones Biomédicas de Canarias (CIBICAN), Universidad de La Laguna (ULL), Avda. Astrofísico F. Sánchez, 2, 38206 La Laguna, Tenerife, Spain; adiazmar@ull.edu.es (A.R.D.-M.); jjfercas@ull.edu.es (J.J.F.); 3 Servicio de Oncología Ginecológica, Unidad Médica de Alta Especialidad, Hospital de Gineco-Obstetricia No. 23, Instituto Mexicano del Seguro Social (IMSS), Avenida Constitución y Félix U. Gómez s/n, Colonia Centro, 64000 Monterrey, Nuevo León, México; davame7@hotmail.com; 4 Departamento de Anatomía Patológica, Unidad Médica de Alta Especialidad, Hospital de Gineco- Obstetricia No. 23, Instituto Mexicano del Seguro Social (IMSS), Avenida Constitución y Félix U. Gómez s/n, Colonia Centro, 64000 Monterrey, Nuevo León, México; oesh10@gmail.com; 5 Centro de Investigación Biomédica del Noreste (CIBIN), Instituto Mexicano del Seguro Social (IMSS), Calle Jesús Dionisio González # 501, Col. Independencia, 64720 Monterrey, Nuevo León, México; carranza60@yahoo.com.mx; 6 División de Investigación en Salud, Unidad Médica de Alta Especialidad, Hospital de Cardiología No. 34, Instituto Mexicano del Seguro Social (IMSS), Av. Lincoln S/N esquina con Enfermera María de Jesús Candia, Col. Valle Verde 2do. Sector, 64360 Monterrey, Nuevo León, México

**Keywords:** laurinterol, *Laurencia*, antitumoral, breast cancer explants, organotypic culture, ex vivo

## Abstract

Macroalgae represent an important source of bioactive compounds with a wide range of biotechnological applications. Overall, the discovery of effective cytotoxic compounds with pharmaceutical potential is a significant challenge, mostly because they are scarce in nature or their total synthesis is not efficient, while the bioprospecting models currently used do not predict clinical responses. Given this context, we used three-dimensional (3D) cultures of human breast cancer explants to evaluate the antitumoral effect of laurinterol, the major compound of an ethanolic extract of *Laurencia johnstonii*. To this end, we evaluated the metabolic and histopathological effects of the crude extract of *L. johnstonii* and laurinterol on Vero and MCF-7 cells, in addition to breast cancer explants. We observed a dose-dependent inhibition of the metabolic activity, as well as morphologic and nuclear changes characteristic of apoptosis. On the other hand, a reduced metabolic viability and marked necrosis areas were observed in breast cancer explants incubated with the crude extract, while explants treated with laurinterol exhibited a heterogeneous response which was associated with the individual response of each human tumor sample. This study supports the cytotoxic and antitumoral effects of laurinterol in in vitro cell cultures and in ex vivo organotypic cultures of human breast cancer explants.

## 1. Introduction

Marine environments are an interesting source of compounds holding a variety of therapeutic properties as a result of the vast diversity of lifeforms inhabiting the oceans. Among them, algae are one of the most prolific sources of bioactive compounds. Unfortunately, over the last two decades, only nine marine-derived drugs were approved for clinical therapy in spite of more than 18,000 new marine compounds described in that time frame. Additionally, it is interesting to see that six out of these nine approved drugs are currently used in cancer therapies [1].

The genus *Laurencia* is one of the richest sources of novel compounds among red algae. It is widely distributed throughout tropical and temperate zones. In particular, *L. johnstonii* is an endemic species of the Gulf of California in Mexico [2], present throughout the year in regions compatible with its temperate biogeographic affinity [3].

As a consequence of its wide distribution, the biological activity of metabolites isolated from *Laurencia* species were tested. Laurinterol (L1) is a brominated sesquiterpene frequently found in *Laurencia* species and mollusks of genus *Aplysia* [4]. Its antibacterial [5,6,7], cytotoxic [7,8], antifouling [9], Na/K ATPase inhibition [10], and insecticidal and repellent [11] properties were reported. Recently, our group reported L1 anti-*Acanthamoeba* activity [12].

Breast cancer is the most common cancer worldwide, impacting 2.1 million women each year, and it is the second highest cause of cancer death in women with 627,000 deaths in 2018 [13]. The most common drugs used for breast cancer therapy include anthracyclines, such as doxorubicin and epirubicin, taxanes, such as paclitaxel and docetaxel, 5-fluorouracil (5-FU), cyclophosphamide, and carboplatin. In most cases, combinations of two or three of these drugs are used [14]. However, there is a need for new anti-cancer agents with fewer and/or less significant side effects, and the effectiveness of available option treatments is still limited. In addition, resistance to chemotherapy is an important problem in breast cancer management, and multidrug resistance (MDR) was observed as a result of cross-resistance to other cytotoxic agents to which patients were never exposed [15]. Drug resistance in cancer can be mediated via different mechanisms, such as alterations affecting cell-cycle dynamics, susceptibility of cells undergoing apoptosis, uptake and efflux of drugs, cellular drug metabolism, intracellular compartmentalization of drugs, or repair of drug-induced damage [15].

As a result of the numerous research group efforts to find new targets and novel anticancer compounds, recently, a number of medical advances improved the treatment options against breast cancer, many of which are geared toward the individual characteristics of the patient and the tumoral tissue. The goal of these kinds of treatments is to be as effective as possible, while keeping minimal side effects and treating only the patients who will benefit from a specific therapy [16,17].

After decades of research, experimental models that are able to correlate the compounds’ activity with their clinical efficacy in humans, as well as to predict an individual response to treatment, are still needed. In this regard, an organotypic culture of tumor-derived slices is a robust model that retains the tumor microenvironment and allows extrapolating the effect of antineoplastic agents in terms of physiology, metabolism, and pharmacokinetics [18]; it is a promising option between two-dimensional (2D) cell culture and pre-clinical trials, while decreasing the high risk of failure in the drug development pipeline and improving the therapeutic response prediction.

Taking into consideration the cytotoxic activity of *Laurencia-*derived metabolites [4,8,12,19,20,21,22], the rapid breast cancer increment, and the need for more effective drugs, we analyzed the antineoplastic effect of L1, the major compound isolated from *L. johnstonii*, using ex vivo organotypic cultures of human breast cancer explants.

## 2. Results and Discussion

### 2.1. Extraction and Isolation

The marine environment is an interesting source of bioactive compounds with uncommon chemical features. Among marine organisms, algae are one of the major sources of new compounds after sponges, microorganisms, and phytoplankton [1]. Red algae of the genus *Laurencia* are considered as one of the richest sources of new secondary metabolites with a huge chemical variation influenced by environmental and genetic factors [4]. The study of endemic organisms, such as *L. johnstonii*, adapted to live under particular environmental conditions, is a significant aspect to consider in the search of potential bioactive secondary metabolites. In a previous work, we observed better bioactivity profiles in extracts of *Laurencia* species collected in the Gulf of California compared to those of species inhabiting the Pacific coast from the Baja California peninsula [23].

**Figure 1:**
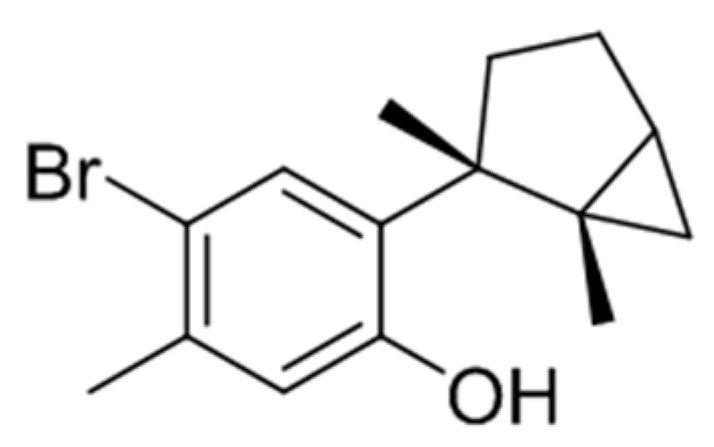
Laurinterol (L1).

L1 (Figure 1) was isolated from the ethanolic extract of *L. johnstonii* [24] and represented 70% of the whole crude extract (CE), a relevant fact if we consider that one of the major challenges in the biodiscovery process is the supply problem [1]. L1 is a brominated sesquiterpene with a cyclolaurane skeleton, widely obtained from *Laurencia* species [4] and recently isolated from *L. johnstonii* [12]. L1 belongs to the significant group of marine haloaryl secondary metabolites, molecules of relevance for their interesting biological activity [25].

### 2.2. Cytotoxicity Assays

**Figure 2:**
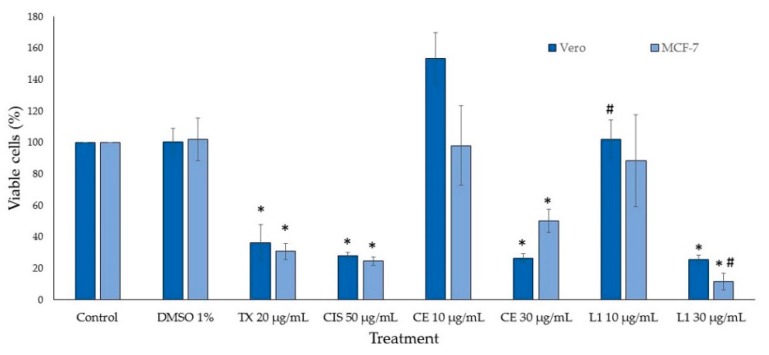
Viability of treated cells. TX: paclitaxel, CIS: cisplatin, CE: crude extract, L1: laurinterol. *n* = 3 ± standard deviation (SD); * *p* < 0.01 compared to the control, # *p* < 0.05 compared to CE.

CE and L1 cytotoxic activity were evaluated against Vero and MCF-7 cell lines. Vero cells are used worldwide as a normal cell line control to assess in vitro cytotoxic effects [26,27], due to its susceptibility to various types of microbes, toxins, and chemical compounds. This cell line is often used in natural product screening assays [28,29,30] and in cancer studies as a normal cell line control group [31]. Cell viability was measured by addition of WST-1 after 24 h of incubation, and it was estimated from the cell population of the control and cell populations after treatments. CE and L1 were assayed at various concentrations and they displayed a dose-dependent inhibition; controls were dimethyl sulfoxide (DMSO), paclitaxel (TX), and cisplatin (CIS). CE showed half maximal inhibitory concentration (IC_50_) values of 26.18 µg/mL and 28.05 µg/mL for Vero and MCF-7 cell lines, respectively, whereas L1 IC_50_ values were 15.68 µg/mL for Vero and 16.07 µg/mL for MCF-7 cells. Figure 2 shows the percentage of cell viability at two different concentrations of CE and L1, one lower (10 µg/mL) and one higher (30 µg/mL) concentration than the IC_50_ values obtained for both cell lines. Both CE and L1 showed similar viability percentage, due to the great amount of L1 in the CE. The cytotoxic effects of *Laurencia* extracts against different cell lines were reported [23,32,33], showing a wide spectrum of toxicity according to the species, geography, and solvent used for the extraction. L1 was also evaluated in cell lines including MCF-7, with similar results to our findings [8]. Additionally, CE induced a notable or discrete hormetic effect at 10 µg/mL in Vero cells or MCF-7 cells; hormesis is a biphasic dose-dependent response that induces an adaptive effect on the cells characterized by low-dose stimulation and high-dose inhibition [34] (Figure 2).

After a period of 24 h, morphological alterations were observed under the microscope in cells incubated with the treatments. Control subject cells in plain growth medium and cells incubated with 1% DMSO appeared to be healthy, displaying well-defined cell–cell junctions and a normal confluent monolayer, while cells treated with antineoplastics (TX and CIS), CE, and L1 became rounder and shrunken, with cell–cell junctions disrupted and detachment from the surface of the plate, denoting cell death (data not shown).

**Figure 3:**
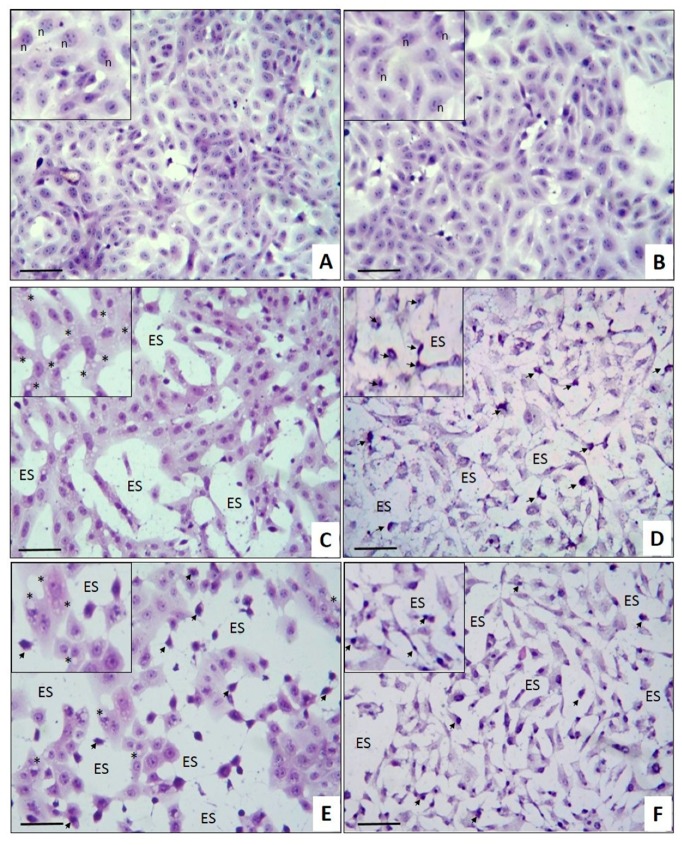
Cytological effects of CE and L1 on MCF-7 cells. Monolayers of MCF-7 human breast cancer cells were incubated with (**A**) cell culture medium, (**B**) 1% dimethyl sulfoxide (DMSO), (C) 30 μg/mL CE, (**D**) 30 μg/mL L1, (**E**) 20 μg/mL TX, and (**F**) 50 μg/mL CIS. Inserts in all images show better morphologic details. (Arrows indicate nuclear pyknosis; n, nucleolus; ES, empty space). Hematoxylin and eosin (H&E) in situ staining. Scale bar: 100 µm. Total magnification: 200×.

The cytological effects of L1 in MCF-7 breast cancer cell monolayers were studied using an in situ hematoxylin and eosin (H&E) staining assay (Figure 3). The untreated control cells (Figure 3A) showed the characteristic cell monolayers irregularly shaped, with well-defined junctions and preserved nucleus–cytoplasm ratio, with a prominent nucleus and more than one nucleolus in each cell. Cells incubated with 1% DMSO (Figure 3B) showed a similar morphology to the untreated cells. In the cells treated with CE (Figure 3C), the cytotoxic effect was characterized by the presence of cytoplasmic vacuoles of different sizes in all cells (asterisks), and by the monolayers’ partial rupture where cytoplasmic junctions can be observed in empty spaces (ES) instead of cells, while their nucleoli became less prominent. In contrast, the cytotoxic effect of L1 (Figure 3D) is notably different than the CE; in this case, it is possible to observe the cell monolayer’s complete rupture, loss of cell morphology, and loss of the nucleus–cytoplasm ratio, as well as cell shrinkage and nuclear pyknosis (arrow). The morphologic and nuclear changes observed in the present study are apoptotic cell death characteristics [35], and they were described in breast cancer cell lines treated with antiproliferative agents [36], extracts [37], and pure compounds, such as curcumin [38]; this also agrees with Kim et al., who reported that a crude extract of *L. okamurai* containing laurinterol can induce apoptosis through a p53-dependent pathway in melanoma cells [22].

The CE cytological effects are similar to those induced by TX (Figure 3E), while L1 induces damage similar to CIS (Figure 3F), suggesting different molecular damage mechanisms. At this level, both chemotherapeutic drugs exhibit a greater antiproliferative effect, as they showed a marked inhibition of monolayer formation. Considering that L1 is the major compound in the CE, it could be expected that the observed cytological effect would also be similar. In order to establish the possible relationship between the CE and L1 mechanism of action with the antineoplastics TX and CIS, additional studies are required. On the other hand, it is also possible that the cytological differences induced by the CE and L1 can be attributed to other minor metabolites present in the CE, such as the bromo-sesquiterpene aplysin, which was also found in the CE of *L. johnstonii* [12] and which was reported as a powerful apoptosis inductor [4,39,40,41]. Other minor compounds isolated from *L. johnstonii* (isolaurinterol, α-bromocuparane, α-isobromocuparane [12], johnstonol [42], and prepacifenol epoxide [43]) also showed cytotoxicity in cell lines [19,20,21,44,45] and murine macrophages [12], with isolaurinterol showing the highest toxicity. With regard to the apoptosis in MCF-7 cells described in this work, further studies are needed to identify the underlying mechanism.

### 2.3. Antitumoral Effect on Breast Cancer Explants

In the last few decades, seaweed chemical profiles revealed a significant number of promising cytotoxic compounds against a wide variety of cancer cell lines. However, in vivo studies with these compounds are limited most likely because they are either scarce in nature or they are too structurally complex for their total synthesis. Furthermore, the majority of these in vivo studies were performed in mice with induced carcinogenesis, and human cancer cell line xenografts in immunocompromised mice, guinea-pigs, and zebrafish, which do not reflect the in vivo situation [45]. Despite these models’ improvements, it is still not possible to predict clinical results [46].

Considering the results described above, and the necessity for more predictive models to identify cytotoxic compounds with high success rates to proceed toward the clinical trial phase, we decided to evaluate the effect of the CE and L1 in a more robust three-dimensional (3D) model that is able to maintain the tumor environment ex vivo. Precision-cut tissue slices represent an intermediate experimental approach between in vivo and in vitro models, which retain histological and three-dimensional structure, with inter- and extracellular interactions, cell matrix components, and metabolic capacity [47]. There are several studies that report ex vivo tumor responses to anticancer drugs in tissue slices in breast [48,49,50,51,52], liver [53], head and neck [54,55], colorectal [56], gastric, esophagogastric [57], lung [58], pancreatic [59,60], prostate, and bladder [61] cancers. Since different types of tumors display different growth and culture characteristics, the aforementioned studies served to standardize a number of differing tumor-slice culture systems; for example, recently, we used breast tumor explants prepared from precision-cut breast slices to study the antitumoral effect of a number of natural compounds [62].

**Figure 4:**
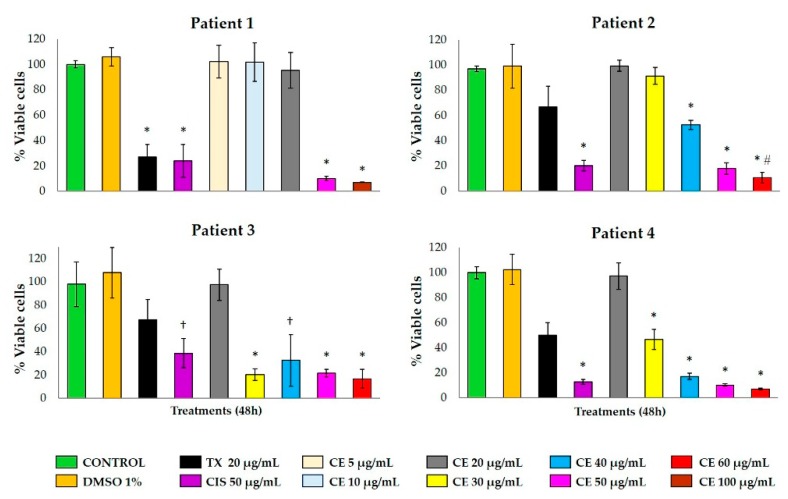
Effect of CE on the metabolic viability of human breast tissue explants. Different concentrations of CE (5–100 µg/mL) were tested in tissue samples from four different patients with breast cancer. CIS (50 μg/mL) was used as a pharmacologic control. Data are expressed as means ± standard deviation (SD). * *p* < 0.01, † *p* < 0.05 compared to the control; # *p* < 0.01 compared to CIS.

**Figure 5:**
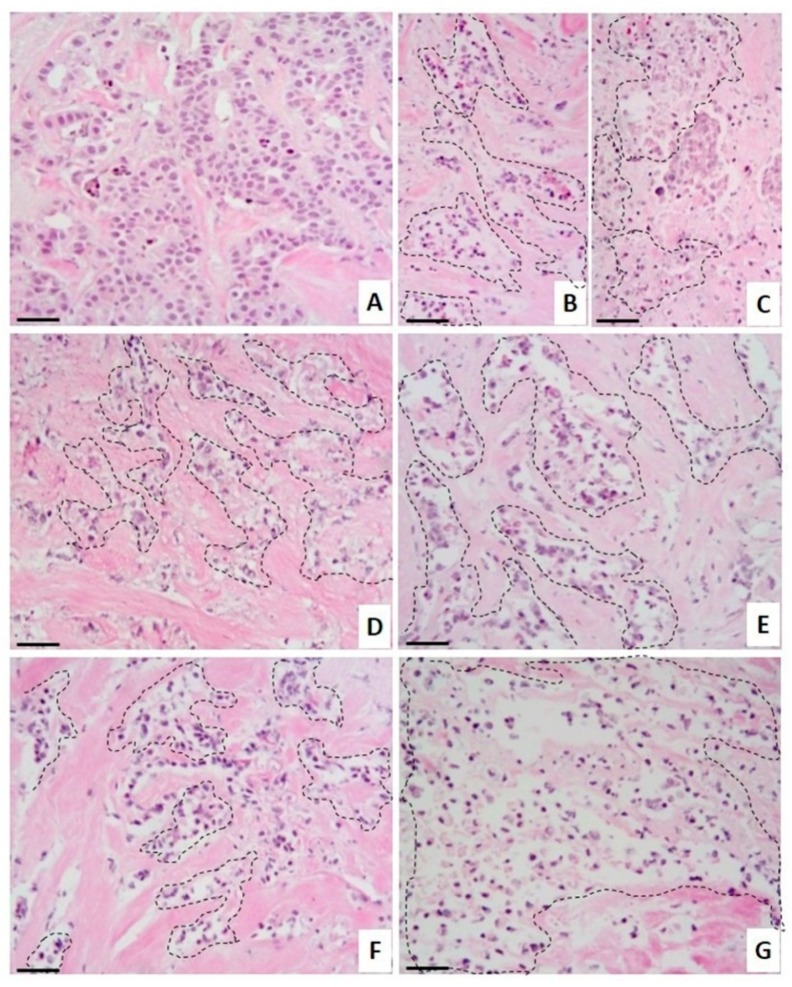
Histopathological analysis of breast tissue explants treated with crude extract (CE). Representative images of CE’s effect on human breast tumor tissue explants. (**A**) Tumor explants not subjected to treatment were incubated in culture medium, or incubated in the presence of (**B**) 20 μg/mL TX, (**C**) 50 μg/mL CIS, and different concentrations of CE: (**D**) 30 μg/mL, (**E**) 40 μg/mL, (**F**) 50 μg/mL, and (**G**) 60 μg/mL. H&E staining. Dotted areas indicate necrosis of the tissue. Scale bar: 100 µm. Total magnification: 100×.

**Figure 6:**
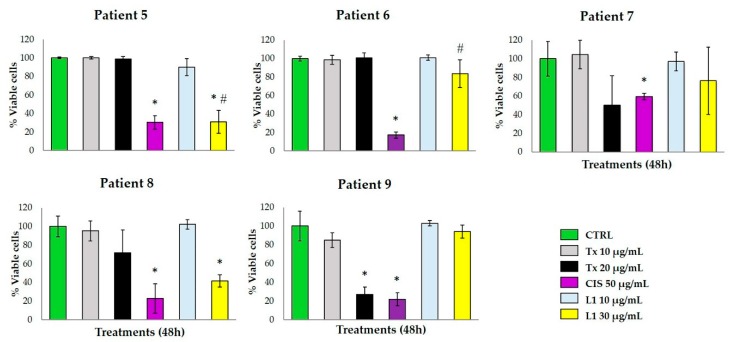
Effect of L1 on the metabolic viability of human breast tissue explants. Tissue explants of human breast tumor from five patients were incubated with DMEM/F12 supplemented medium and treated with L1 at 10 and 30 μg/mL. TX (20 μg/mL) and CIS (50 μg/mL) were used as pharmacological controls. Data are expressed as means ± standard deviation (SD). * *p* < 0.05 compared to the control; # *p* < 0.05 compared to TX (20 μg/mL).

**Figure 7:**
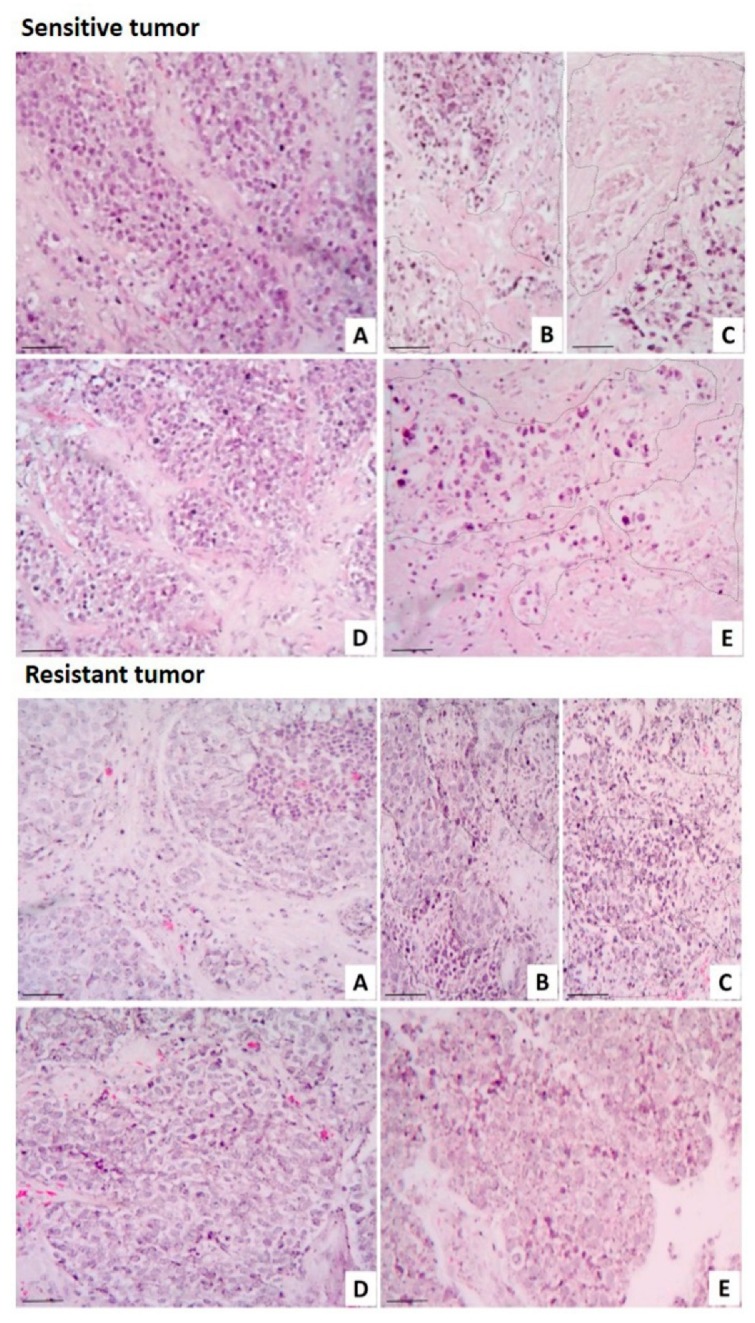
Human breast tumors with different responses to laurinterol (L1). Representative images of human breast tumors that were sensitive (upper panel) and resistant (lower panel) to L1. (**A**) Control tissue explants were grown in culture medium; treated explants were incubated in the presence of (**B**) TX, (**C**) CIS, (**D**) 10 μg/mL L1, and (**E**) 30 μg/mL L1. Dotted areas indicate necrotic tissue. H&E staining. Scale bar: 100 µm. Total magnification: 100×.

In order to evaluate the effect of CE and L1, ex vivo cultures of breast cancer explants were obtained after surgery from nine breast cancer patients with histopathological diagnosis of infiltrating ductal/lobular adenocarcinoma. Clinical and histopathological data of the subjects are summarized in Table 1. Four tumor samples were used in four independent assays to evaluate the effect of the CE (Figure 4; Figure 5), while the other five samples were used to test L1 (Figure 6 and Figure 7).

A range from 40 to 64 breast cancer explants with a 4-mm diameter and 250–300-µm thickness were obtained from each tumor, and they were incubated for 48 h at 37 °C with different treatments (as referred to in the tumor explant treatment section). Since human tumor tissue is not always available in same quantity/quality to prepare tissue explants, we decided to use a CE dose range from small to higher concentrations (5–100 µg/mL) in explants from Patient 1. On the other hand, because the crude extract did not show activity at small concentrations, we re-adjusted the concentration (20–60 µg/mL) for the next three patients’ explants.

The explants’ metabolic activity was measured with the Alamar Blue™ assay. CE-treated breast tumor samples showed a considerable reduction in tumor metabolic viability at concentrations greater than 30 µg/mL, similar to the effect of the antineoplastic drug CIS (Figure 4). Patients 2 to 4 showed partial resistance to the metabolic activity reduction with TX. According to the histopathological analysis (Figure 5), untreated tumor tissue was preserved (Figure 5A) and both antineoplastics, TX (Figure 5B) and CIS (Figure 5C), induced marked necrosis areas in the neoplastic cells (dotted areas), although CIS exhibited a more marked necrotic effect. CE also induces necrosis, which increased gradually depending on its concentration (Figure 5D–G).

The metabolic activity of breast tumor explants treated with L1 at 30 μg/mL concentration provoked a marked viability reduction in patients 5 and 8, with a higher effect on the metabolic viability of the tumor tissue compared to TX. Samples from patients 7 and 9 were resistant to the L1 antineoplastic effect, and patient 6 was resistant to TX and L1. All five samples were sensitive to the antineoplastic CIS (Figure 6). The resistance to the antineoplastic drugs used as pharmacologic controls in the present study is possibly due to a reduced intracellular drug concentration of taxol [16], and a reduction of cellular drug uptake of cisplatin by the cancerous cells [17].

As a complement for the in vitro and the metabolic viability assays, histopathological analyses were performed. Histomorphologic findings from the untreated breast tumor explants (control) showed that the tissue typical architecture was preserved under culture conditions (Figure 7A). As expected, the antineoplastic drugs induced necrosis in the neoplastic cell groups to both the sensitive samples and the resistant ones (Figure 7B,C). No tissue damage was observed in the sensitive tumor sample incubated with L1 at 10 μg/mL; however, marked necrotic cell death was induced with the 30 μg/mL concentration. On the other hand, no toxic effects were observed on the resistant tumor samples incubated with 10 μg/mL and 30 μg/mL L1 (Figure 7D,E). Additionally, we evaluated the possible synergistic effect between L1 and TX; however, no effect was detected in either the metabolic activity or the histopathological analysis (data not shown).

The tumor samples used in the present work were classified according to the main breast cancer molecular subtypes as luminal A, luminal B, HER2 non-luminal, and basal-like. As it is known in breast cancer, the overexpression of the estrogen receptor (ER), the progesterone receptor (PR), and oncogenes such as *HER2*, among other biomarkers, are routinely checked with immunohistochemistry, and they are associated with prognosis and prediction of treatment response. The best-characterized breast cancer subtypes are designated as luminal A, luminal B, HER2, and basal-like [63,64].

Our results suggest that there is no predictive relationship between the molecular subtype and the treatment response; the existence of different breast cancer subtypes within a single tumor [65] may account for these results. Despite the samples included in this study belonging to the same histological type, the differential response to the treatments may be due to each patient’s complex inherent response, and the heterogeneity inside each tumor sample. Similar results were reported by van der Kuip et al., who found a wide treatment response gamma ranging from strong resistance to intermediate response, sensible to the antineoplastic, when exposing breast cancer slices from 10 patients diagnosed with invasive ductal type to different taxol doses [48].

In addition to intratumoral heterogeneity, our results exhibit how patient response to treatment varies on a case-by-case basis. Therefore, assays aimed toward predicting whether or not an individual tumor responds to cancer treatments are needed, and we believe that tumor-derived organotypic cultures provide a suitable approach to deal with said requirement [50,54,56,57,60,62], since they comprise various cells that are collectively important for tissue homeostasis, as well as tumor response [66]. As it was proven in the present study, cell cultures are a helpful approach as a first screening option due to their simplicity. Nevertheless, overcoming the unrealistic monolayer culture’s growth conditions remains a necessity and an interesting opportunity for future studies.

## 3. Materials and Methods

### 3.1. General Experimental Procedures

Optical rotations were measured in CH_2_Cl_2_ on a PerkinElmer 241 polarimeter (Waltham, MA, USA) using an Na lamp. NMR spectra were recorded on a Bruker AVANCE 500 MHz (Bruker Biospin, Fällanden, Switzerland). NMR spectra were obtained dissolving samples in CDCl_3_ (99.9%) and chemical shifts are reported relative to solvent (δ_H_ 7.26 and δ_C_ 77.0 ppm) and TMS as an internal pattern. HR-ESI-MS data were obtained on an LCT Premier XE Micromass spectrometer (Waters, Milford, CT, USA). Thin-layer chromatography (TLC; Merck, Darmstadt, Germany) was visualized by spraying with cobalt chloride reagent 2% (10% sulfuric acid) and heating.

Paclitaxel, cisplatin, DMEM/F12 medium, fetal bovine serum, gentamicin, penicillin–streptomycin, and Alamar Blue™ were obtained from Invitrogen (Gran Island, NY, USA). The reagents for general use were purchased from Sigma-Aldrich (St. Louis, MO, USA).

### 3.2. Biological Material

*L. johnstonii* was collected by hand during the summer off the coast of Baja California Sur, Mexico (24°21’10.8” north (N), 110°16’58.8” west (W)). A voucher specimen (code 13-003) was deposited at the Herbarium of the Laboratory of Marine Algae of the Interdisciplinary Center of Marine Science (CICIMAR) and it was identified by Dr. Rafael Riosmena Rodríguez from the Autonomous University of Baja California Sur (UABCS, La Paz, B.C.S., México).

### 3.3. Extraction and Isolation

Washed and dried specimens of *L. johnstonii* were crushed and extracted with EtOH for three days at 25 °C under gentle agitation. The dissolvent was replaced three times and the ethanol was combined and filtered through a Whatman no. 4 filter paper. Solvent was removed using a rotatory vacuum evaporator. Then, 10 g of the resulting extract was chromatographed in Sephadex LH-20 (500 × 70 mm, CH_3_OH 100%) to obtain five fractions. Fraction 4 (1 g) was separated in a Silicagel open column (25 × 5 cm) using a stepwise gradient of *n*-hexane–ethyl acetate to yield pure L1 (*n*-hexane 100%, 707.9 mg).

Laurinterol (**L1**): C_15_H_19_BrO, HR-ESI-MS *m/z* 293.0531 [M − H]^−^ (calculated C_15_H_18_O^79^Br, 293.0541), 295.0518 [M − H]^−^ (calculated C_15_H_18_O^81^Br, 295.0521) $[\alpha {]}_{D}^{25}$ + 17 (*c* 0.15, CH_2_Cl_2_) ^1^H NMR (CDCl_3_) δ 0.55 (1H, dd, *J* = 7.9, 4.8 Hz, H-12), 0.58 (1H, t, *J* = 4.6 Hz, H-12), 1.15 (1H, dt, *J* = 8.1, 4.3 Hz, H-3), 1.28 (1H, m, H-5), 1.32 (3H, s, H-13), 1.41 (3H, s, H-14), 1.66 (1H, dd, *J* = 12.3, 8.0 Hz, H-4), 1.95 (1H, tdd, *J* = 12.3, 8.1, 4.4, H-4), 2.09 (1H, dd, *J* = 13.2, 8.1 Hz, H-5), 2.29 (3H, s, H-15), 5.26 (1H, br, s, 7-OH), 6,61 (1H, s, H-8), 7.61 (1H, s, H-11); ^13^C NMR (CDCl_3_) δ 16.2 (C-12), 18.6 (C-13), 22.2 (C-14), 23.5 (C-15), 24.4 (C-3), 25.3 (C-4), 29.6 (C-2), 35.9 (C-5), 114.9 (C-10), 118.8 (C-8), 132.3 (C-11), 134.0 (C-6), 135.9 (C-9), 153.3 (C-7).

### 3.4. Cell Culture

Vero (kidney normal epithelial cell line, ATTC^®^ number CCL-81™, Manassas, VA, USA) and MCF-7 (mammary adenocarcinoma cell line, ATCC^®^ number CCL-2™) cells were grown in MEM and DMEM/F12 medium (1:1 mixture containing 2.5 mM l-glutamine, 15 mM HEPES, 0.5 mM sodium pyruvate, 17.5 mM d-Glucose, and 1.2 g/L sodium bicarbonate) supplemented with 10% (*v*/*v*) FBS and penicillin–streptomycin, respectively. Cultures were routinely maintained at 37°C in a humidified 5% CO_2_ atmosphere.

### 3.5. Cytotoxicity Activity

The assays were performed in 96-well microplates, containing 15,625 cells/cm^2^ (5 × 10^3^ cells/well) and exposed to different concentrations of the extract and the pure compound for 24 h. Cell viability was measured by WST-1 addition and 90 min of incubation. The absorbance was measured at 450 nm, and the concentration of the samples that inhibited 50% of the cell growth (IC_50_) was calculated. Three experiments were performed in triplicate. DMSO (1%), CIS (50 μg/mL), and TX (20 μg/mL) were used as controls. TX concentration was chosen on basis of our previous work [62]. Cisplatin concentration was selected by testing several doses in MCF-7 cells and breast cancer explants.

### 3.6. In Situ H&E Staining of MCF-7 Cells

To elucidate partially the damage mechanism induced by both CE and L1, we performed an in situ staining that allowed observing, at a cellular level and in greater detail, the toxic effects induced by different compounds. The MCF-7 breast cancer cell line (52,632 cells/cm^2^ = 5 × 10^5^ cells/well) was cultured on sterile glass coverslips contained in six-well microplates and incubated at 37 °C with 5% CO_2_ for 24 h. Afterward, the cultures were incubated for additional 24 h in the presence of culture medium (untreated control), 1% DMSO (solvent control), 30 μg/mL CE, and 30 μg/mL L1. The antineoplastic drugs TX and CIS (20 and 50 μg/mL, respectively) were used as pharmacological control references. After the incubation time with the treatments, the cells were fixed and stained in situ by H&E staining, and permanent preparations were made, and they were observed with a Zeiss Axiostar Plus brightfield microscope (Jena, Germany). Representative photographs of all treatments were obtained with a 5.0 MP Moticam camera (Richmond, BC, Canada).

### 3.7. Tumor Samples

The approval to work with human tissues was obtained from the Institutional Review Board (Instituto Mexicano del Seguro Social, Coordinación de Investigación en Salud. R-2014-785-022,). With previous informed consent, infiltrating ductal/lobular adenocarcinoma samples were obtained from nine patients undergoing mastectomy or excisional biopsy for the carcinoma remaining removed as surgical waste once sufficient tissue was assured for clinical diagnosis at the Hospital de Ginecología y Obstetricia (UMAE # 23, Monterrey, Nuevo León, México) from the Instituto Mexicano del Seguro Social (clinical and histopathological data are described in Table 1). Tissues were collected in cold serum-free DMEM/F12 medium and transported at 4 °C to the laboratory for immediate processing.

### 3.8. Preparation of Slices and Explants from Breast Cancer

Breast cancer human tissue slices of 4 mm diameter and 250–300 µm thickness were obtained using the Krumdieck tissue slicer (Alabama Research & Development, Munford, AL, USA) with constant flow of KB buffer at 4 °C gassed with carbogen gas as described previously [62]. In total, 40 to 64 breast tumor explants were prepared and placed in 24-well microplates containing DMEM/F12 supplemented medium with 10% (*v/v*) FBS, 25 mM D-glucose, 1% ITS (insulin-transferrin-selenium), 1 mM sodium pyruvate, plus penicillin–streptomycin, and incubated for 1 h at 37 °C, 5% CO_2_, with agitation at 30 rpm. The interval between the tumor resection and the explant incubation was no more than 2 h.

### 3.9. Treatment of Tumor Explants

After pre-incubation, in order to confirm the tumor samples’ viability, control explants (0 h) were placed in 24-well microplates containing 1 mL of DMEM/F12 supplemented medium and incubated for 4 h at 37 °C, 5% CO_2_/95% air, with agitation at 30 rpm. Depending on the explants’ availability, 40 to 64 explants were cultured per assay, with four to six explants per treatment or experimental condition. Treated explants were incubated for 48 h at 37°C, 5% CO_2_, with agitation at 30 rpm with the following compounds: 20 µg/mL TX, 50 µg/mL CIS, 5–100 µg/mL CE, 10 and 30 µg/mL L1, and 10 µg/mL L1 + 10 µg/mL TX. Controls were medium and 1% DMSO.

### 3.10. Alamar Blue™ Viability Assay

After 48 h of incubation with the compounds, as well as the culture medium (control), the explants were incubated for an additional 4 h with 10% Alamar Blue™ in 500 µL of DMEM/F12 supplemented medium at 37 °C in the conditions described earlier. Afterward, 100 µL was collected from each sample and transferred to a 96-well microplate to read fluorescence at 530-nm excitation/590-nm emission wavelengths. The percentage of viability relative to control was obtained by calculating the percentage of Alamar Blue™ reduction per explant [62].

### 3.11. Histopatological Analysis

After incubation with the treatments, the breast cancer explants were fixed in 10% neutral formalin and embedded in paraffin using conventional histological techniques. Tissue sections of 4 µm were prepared on a microtome and mounted on glass slides. Afterward, the slides were deparaffinized and stained with hematoxylin and eosin (H&E). The stained preparations were observed by a pathologist using a Zeiss Axiostar Plus Brightfield microscope, and photographs were obtained with a 5.0 Moticam camera. Histopathological analysis was performed by two independent pathologists, and the morphological parameters analyzed included necrosis, viable/damaged tumor cells, and inflammation.

### 3.12. Statistical Analysis

Statistical analysis was performed with SPSS version 20.0 software. Quantitative data were expressed as means and standard deviation. Differences in continuous variables with normal distribution were analyzed with Student’s *t*-test or the Mann–Whitney *U* test for non-normal distributions.

## 4. Conclusions

To our knowledge, this is the first study of a marine natural product in human breast tumor-derived organotypic culture, which supports the known in vitro cytotoxic activity of laurinterol in a more complex system. Our results show a dose-dependent inhibition of the metabolic activity and morphological changes characteristic of apoptotic cell death in cells treated with the crude extract and laurinterol. Meanwhile, breast cancer explants treated with the extract showed reduced metabolic viability and necrosis areas, while explants treated with laurinterol exhibited a heterogeneous response associated with the individual response of each tumor sample. This study emphasizes the importance of the 3D culture of human cancer tissue slices or tissue explants to improve the selection of new therapeutic options from different sources before preclinical studies, as well as a functional drug testing in personalized oncology, enabling the prediction of the interpersonal tumor response.

## Figures and Tables

**Table 1:** Clinical and histopathological subject data.

Patient	Age	^1^ CS (grading)	^2^ HT	^3^ TS	^4^ ER	^5^ PR	^6^ HER2	^7^ MC
1	38	T2N1MO (IIIA)	ID	3	(+)	(+)	(-)	LA
2	54	T4dN1M0 (IIIB)	ID	5	(+)	(+)	(-)	LA
3	46	T3N1M0 (IIIA)	ID	4	(-)	(-)	(+)	HER2
4	63	T2N0M0 (IIA)	ID	3	(-)	(-)	(+)	HER2
5	61	T2N0M0 (IIA)	ID	4	(-)	(-)	(+)	HER2
6	42	T4bN0M0 (IIIB)	ID	4	(-)	(-)	(-)	BL
7	74	T4bN0M0 (IIIB)	IL	4	(+)	(+)	(-)	LA
8	39	T2N2M0 (IIIB)	ID	5	(-)	(-)	(-)	BL
9	57	T3N1M0 (IIIA)	IL	5.5	(+)	(+)	(-)	LA

^1^ Clinical stage; ^2^ histologic type; ^3^ tumor size (cm); ^4^ estrogen receptor; ^5^ progesterone receptor; ^6^ HER2 status; ^7^ molecular classification. Patient age in years; ID: infiltrating ductal; IL: infiltrating lobular; LA: luminal A; BL: basal-like.
